# Expression mediated by three partial sequences of the human tyrosine hydroxylase promoter *in vivo*

**DOI:** 10.1038/mtm.2016.62

**Published:** 2016-09-21

**Authors:** Anne-Sophie Rolland, Tatyana Kareva, Olga Yarygina, Nikolai Kholodilov, Robert E Burke

**Affiliations:** 1 Department of Neurology, Columbia University, New York, NY, USA; 2 Department of Pathology and Cell Biology, Columbia University, New York, NY, USA

## Abstract

The use of viral vectors to transfect postmitotic neurons has provided an important research tool, and it offers promise for treatment of neurologic disease. The utility of vectors is enhanced by the use of selective promoters that permit control of the cellular site of expression. One potential clinical application is in the neurorestorative treatment of Parkinson’s disease by the induction of new axon growth. However, many of the genes with an ability to restore axons have oncogenic potential. Therefore, clinical safety would be enhanced by restriction of expression to neurons affected by the disease, particularly dopamine neurons. To achieve this goal we have evaluated *in vivo* three partial sequences of the promoter for human tyrosine hydroxylase, the rate limiting enzyme in catecholamine synthesis. All sequences induced expression in dopamine neurons. None of them induced expression in glia or in nondopaminergic neurons in striatum or cortex. We conclude that these sequences have potential use for targeting dopamine neurons in research and clinical applications.

## Introduction

The use of cell selective promoters to regulate the expression of foreign genes delivered by viral vectors offers many advantages for both research and clinical applications. In the research setting, for example, cell specific promoters have found numerous uses to enhance the specificity of optogenetic approaches.^1^ In the clinical realm promoters with specificity have the potential to restrict the cellular site of expression and thereby limit off-target effects. For example, we have recently shown that adult dopamine neurons of the mesencephalon, which are predominantly affected in human Parkinson’s disease (PD), can be induced to grow new axons following transduction with constitutively active forms of the kinase Akt or the GTPase hRheb.^2,3^ These observations provide an experimental basis for a new approach to neurorestoration for PD. However, both of these molecules are known to participate in oncogenesis. Therefore, to administer them intracerebrally under the control of a general mammalian promoter for clinical treatment would carry significant risk. For clinical therapeutics the safety of this approach would be greatly enhanced by restricting the expression of these molecules to the desired cellular targets. In the case of PD, for the treatment of its motor manifestations, the relevant population is the dopamine neurons of the substantia nigra pars compacta (SNpc).

The promoter for tyrosine hydroxylase (TH), the rate limiting enzyme in catecholamine biosynthesis, has received extensive prior characterization for achieving selective expression in these neurons. In addition, it provides a number of options for promoter sequences that can be accommodated by the space limitations of an adeno-associated viral (AAV) vector (≤ 4.7 kb). For the purposes of characterizing the human TH promoter (hTHp) sequences that could ultimately be used in gene therapies for human PD, and for transduction of human induced pluripotent stem cells (iPSCs) derived to acquire the dopamine neuron phenotype, we selected three published hTHp sequences that could be accommodated by AAV vector packaging. A 522 bp sequence derived from the 5′ region of the human *TH* gene was identified by Coker *et al*. and shown to be sufficient to drive reporter expression with selectivity for a neuronal cell line.^4^ This sequence was subsequently used to mediate expression in a human neuroblastoma cell line.^5^ Gardaneh and colleagues demonstrated that a striking synergy can be achieved by use of both 5′ and 3′ flanking sequences of the hTHp.^6^ A 3.3 kb sequence derived from the 5′ region of the hTHp was found by Kim and colleagues to drive reporter expression in human neuroblastoma cells but not in immature human neural stem cells.^7^

The performance of a promoter can be expected to be modified by the cellular context in which it acts. Therefore, since our ultimate goal is to identify promoter sequences that will retain strength and specificity in the *in vivo* context, we evaluated each of these sequences in living mice by AAV-mediated transduction of the SN and control brain regions.

## Results

An initial assessment of each of the three hTHp constructs at 4–6 weeks following injection of the SN revealed no toxicity to dopaminergic neurons. Sections immunostained for TH with thionin counterstain revealed no evidence of dopamine neuron loss or an inflammatory response (data not shown).

**Figure 1:**
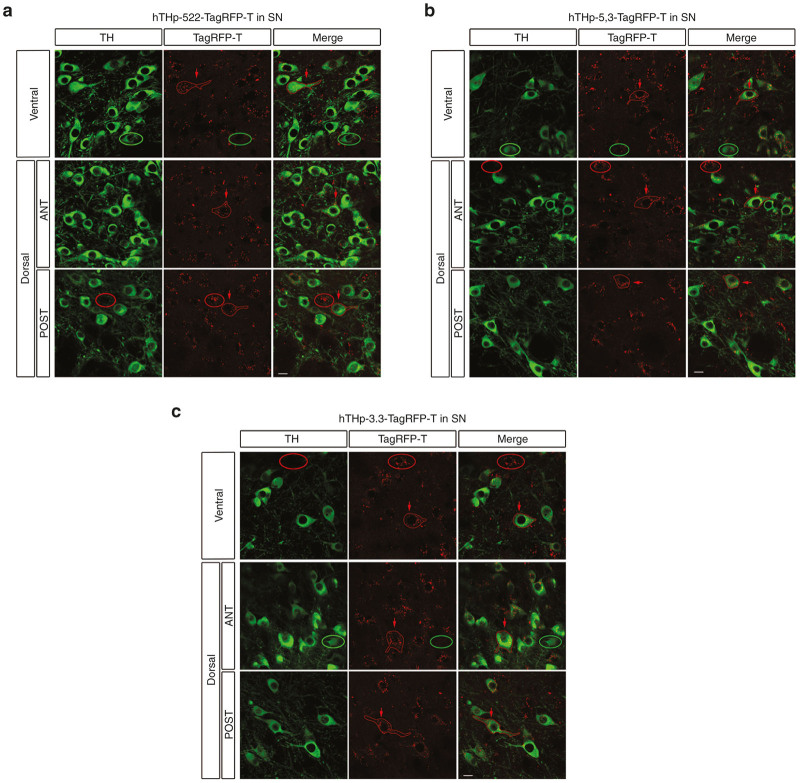
All three partial sequences of the human TH promoter (hTHp) induce expression of the reporter TagRFP-T in neurons in substantia nigra pars compacta (SNpc). Four mice were studied for each sequence and representative images from a single mouse are shown. Two dorsal-ventral planes of horizontal sections were examined to sample the SNpc. For the dorsal plane we examined both anterior and posterior regions. Expression of TagRFP-T (delineated by red contours) was observed in dopaminergic neurons, identified by TH immunofluorescence staining (in green). Red arrows indicate examples of TagRFP-T-positive expression in dopamine neurons in each SNpc region. Green ellipses indicate neurons expressing TH but not TagRFP-T. Red ellipses indicate neurons expressing TagRFP-T but not TH. The 522 bp fragment of the hTHp is shown in (**a**), the combination of 5′ and 3′ flanking region of the hTHp in (**b**), and the 3.3 kb fragment in (**c**). Bar: 10 µm.

**Figure 2:**
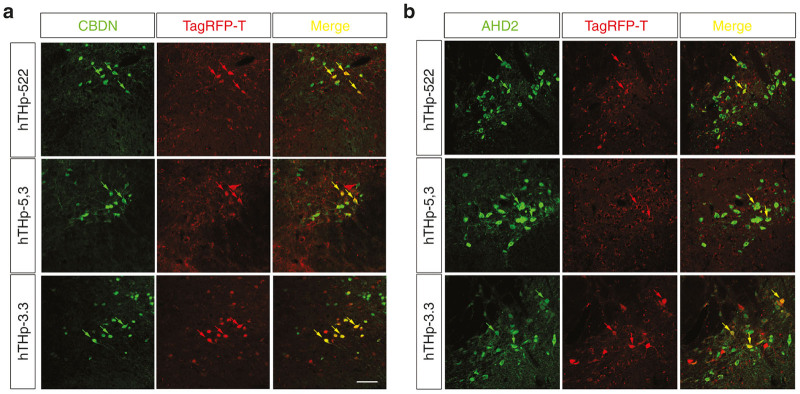
All three partial sequences of human TH promoters (hTHps) induce expression of the reporter TagRFP-T in the ventral and dorsal tiers of the substantia nigra pars compacta (SNpc). To identify expression mediated by each promoter in subtypes of dopaminergic neurons, immunofluorescence for (**a**) calbindin (CBDN), a marker for SNpc dorsal tier, and (**b**) aldehyde dehydrogenase 1A1 (AHD2), a marker for SNpc ventral tier, were performed. Four mice were studied for each sequence and representative images from a single mouse are shown. Yellow arrows indicate double-labeled neurons expressing the reporter TagRFP-T (red arrows) and either CBDN or AHD2 (green arrows). All three hTHp mediate expression in both subtypes of dopaminergic neurons in the SNpc. Bar: 50 µm.

A qualitative assessment of TagRFP-T expression mediated by each of the hTHp promoter sequences demonstrated that each was capable of mediating expression in dopamine neurons of the SN (Figure 1). However this analysis also revealed that each of the promoter sequences mediated expression in non-TH-positive profiles, and, conversely, failed to express in some TH-positive neurons, thus demonstrating limitations on the specificity and sensitivity for each sequence. The presence of reporter expression in some dopamine neurons but not others led us to question whether selective expression may be related to the presence of dopamine neuron subtypes within the SN.^8,9^ One subtype lies predominantly in the dorsal tier of the SNpc and expresses calbindin; the other resides in the ventral tier and preferentially expresses class 1 aldehyde dehydrogenase (AHD2).^10^ Immunofluorescence for these two markers was performed in order to analyze any possible preferential expression by any of the promoter sequences in either of these two subpopulations (Figure 2). Following injection of the SN with each of the three promoter constructs we observed expression of TagRFP-T in both calbindin and AHD2-positive neurons with qualitatively similar abundance (Figure 2). Thus, we conclude that patterns of staining for each of the sequences are not due to selective expression in dopaminergic neuron subtypes.

**Figure 3:**
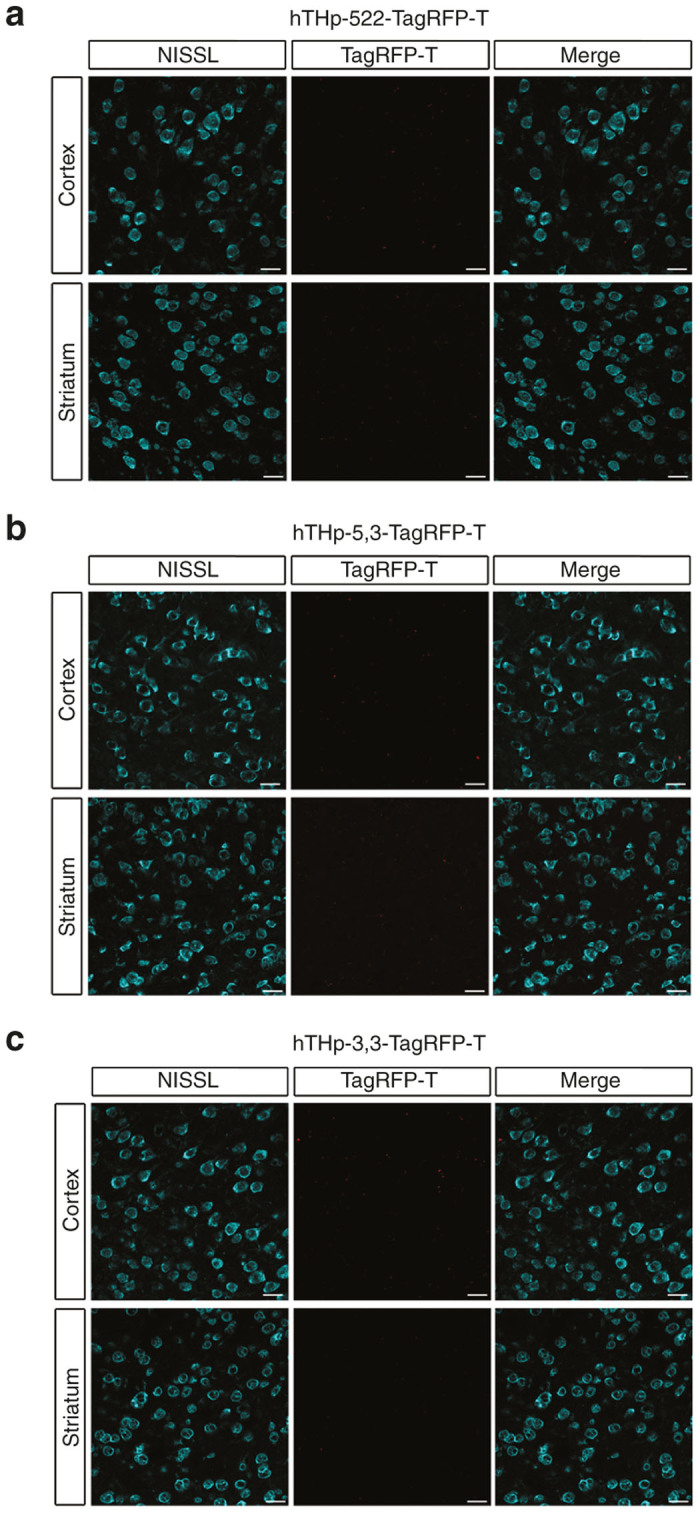
None of the three partial sequences of the human TH promoter (hTHp) induce expression of the reporter TagRFP-T in nondopaminergic regions. To evaluate the specificity of the three sequences, hTHp-522-TagRFP-T (**a**), hTHp-5,3-TagRFP-T (**b**), and hTHp-3.3-TagRFP-T (**c**) were injected into two regions that contain predominantly nondopaminergic neurons: cortex and striatum respectively. A fluorescent Nissl stain was performed in order to visualize neurons in the vicinity of the adeno-associated viral (AAV) injection. Three to four mice were studied for each sequence and representative images from a single mouse are shown. No TagRFP-T positives neurons were observed in any of these regions as shown in the panels. Bar: 25 µm.

In view of the expression of the reporter in non-TH positive neurons in the SN, we next sought to determine the specificity of the promoter sequences in regions where dopamine neurons are not ordinarily found. For this experiment we injected either the striatum or the cortex with each of the three vectors. At 6 weeks after these injections no reporter expression was observed for any of the sequences in either of these regions, confirming specificity of expression (Figure 3).

**Figure 4:**
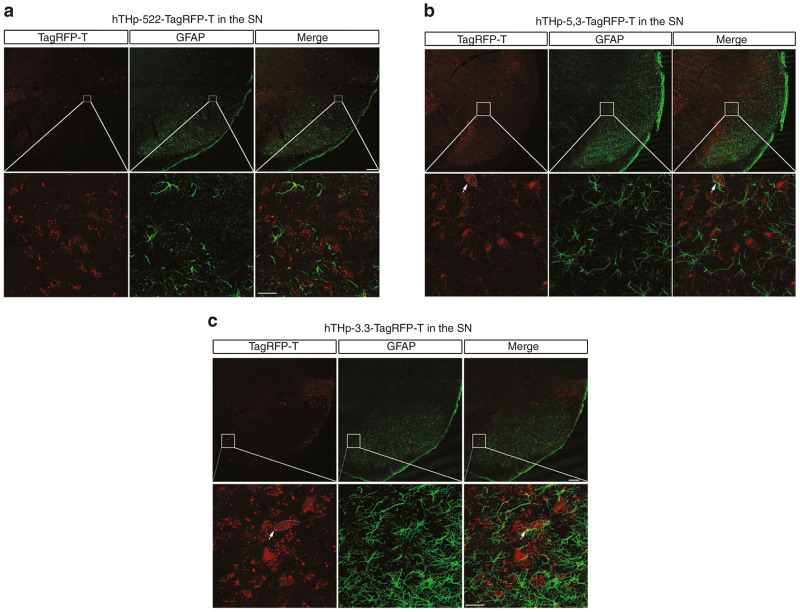
None of the three partial sequences of the human TH promoter (hTHp) induce expression of the reporter TagRFP-T in glial cells. To evaluate the specificity of the three sequences for neuronal expression, hTHp-522-TagRFP-T (**a**), hTHp-5,3-TagRFP-T (**b**), and hTHp-3.3-TagRFP-T (**c**) were injected into the substantia nigra (SN) and immunofluorescence for glial fibrillary acidic protein (GFAP) was performed. Four mice were studied for each sequence and the entire SN was scanned. Representative images from a single mouse are shown. No TagRFP-T expression was observed in glial cells for any of these tyrosine hydroxylase (TH) promoters. For each promoter, GFAP immunofluorescence and TagRFP-T fluorescence within the regions outlined by the squares is shown at higher magnification in the panels below. White arrows indicate a TagRFP-T-positive cell adjacent to a GFAP-positive glial cell without double-labeling. Bar: 100 µm (upper panels), 25 µm (lower panels).

In the evaluation of nondopaminergic staining in the SN it is especially important to determine whether at the cellular level any expression is observed in nonneural cells such as glia, as they could be a particularly important site of off-target effects. We therefore examined reporter expression in astrocytes that had been identified by immunofluorescent staining for glial fibrillary acidic protein (GFAP). For each of the three partial human promoter sequences we were unable to identify any reporter expression in glia (Figure 4).

**Figure 5:**
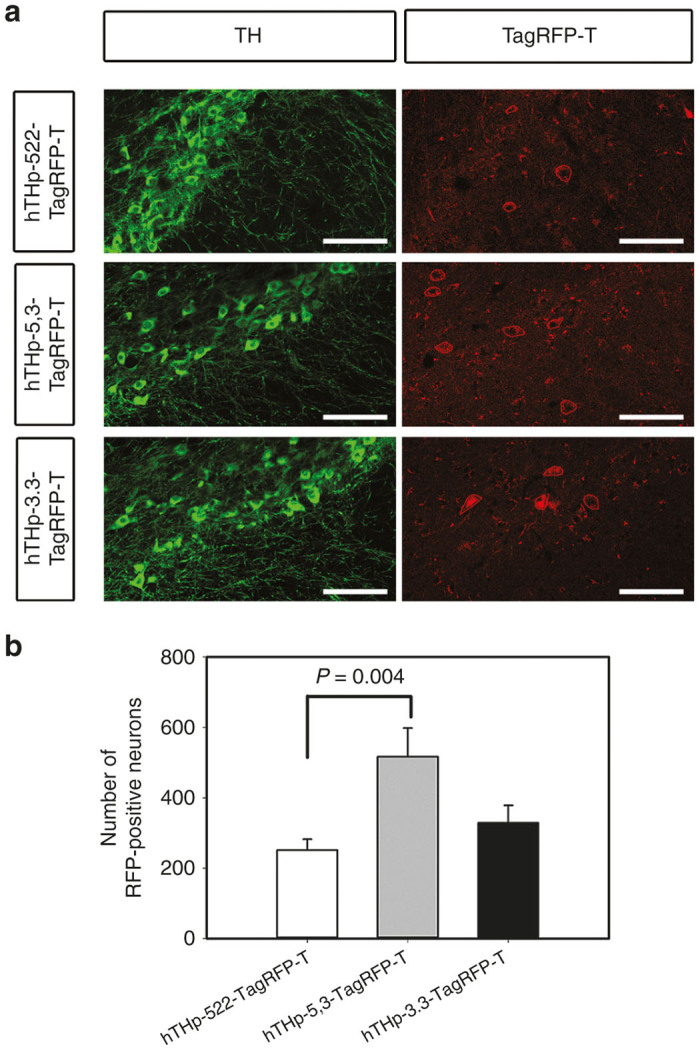
Quantification of the number of neurons expressing the reporter TagRFP-T in the substantia nigra (SN) by three partial sequences of human TH promoter (hTHp). To assess the level of expression mediated by each promoter the number of TagRFP-T-positive cells in the entire SN was determined. To delineate the entire SN, immunofluorescence staining for tyrosine hydroxylase (TH) was performed (**a**). The red contours in the RFP panels show representative examples of TagRFP-T-positive neurons that were counted. (**b**) Quantification of the number of neurons expressing the reporter TagRFP-T shows that the greatest number is achieved by hTHp-5,3-TagRFP-T (517 ± 81.8; *N* = 4), followed by hTHp-3.3-TagRFP-T (328.8 ± 50.2; *N* = 4) and then by hTHp-522-Tag-RFP-T (251.5 ± 30.7; *N* = 12). The hTHp-522-TagRFP-T group contains more animals because the initial experiment showed more variability than the other groups, so it was repeated twice. All data is included in the analysis. There is a statistical difference between the number of TagRFP-T-positive neurons expressed by hTHp-5,3 and by hTHp-522 (*P* = 0.004, one-way analysis of variance (ANOVA)). This result was achieved by hTHp-5,3 in spite of being injected at a lower viral genome dose. Bar: 100 µm.

In view of the similarity in the performance of these hTHp sequences in these qualitative assessments, we undertook to quantitatively evaluate their strength, specificity, and sensitivity of expression in the SN. We first addressed the abundance of reporter expression in the whole SN following injection of each of the three constructs. The hTHp-5,3-TagRFP-T construct showed the highest number of TagRFP-T-positive neurons (517 ± 81.8), and a statistically significant difference in comparison to hTHp-522-TagRFP-T which had the lowest number of TagRFP-T-positive neurons (251.5 ± 30.7; *P* = 0.004, one-way analysis of variance) (Figure 5b). While there was a trend for hTHp-5,3-TagRFP-t to be higher than hTHp-3.3-TagRFP-T, this difference did not achieve significance.

In order to evaluate the sensitivity and specificity of expression in dopamine neurons of the SN, we quantified the total number of TH-positive neurons, TagRFP-T/Nissl-positive neurons and TagRFP-T/Nissl/TH neurons. The three promoter sequences showed only modest levels of sensitivity for detection of dopamine neurons in the SN (Table 1). The most effective construct was hTHp-522-TagRFP-T which achieved expression in 76% of dopamine neurons within the whole SN and a high of 93% within the SN pars reticulata (SNpr). Surprisingly, the two larger promoter sequences had much lower levels of sensitivity at 33 and 43% respectively for hTHp-5,3-TagRFP-T and hTHp-3.3-TagRFP-T. Given the lack of expression by any of these sequences in nondopaminergic regions such as cortex and striatum it was surprising to find such low levels of specificity in the SN ranging from 20% for hTHp-522-TagRFP-T to 17% for hTHp-5,3-TagRFP-T.

## Discussion

Prior studies of these hTHp sequences have been exclusively *in vitro*. We demonstrate here that each of these sequences has the ability to mediate reporter expression in dopamine neurons of the SN *in vivo*. Therefore, they have potential use in clinical applications.

Our primary goal in assessing these sequences was to determine their specificity, in order to ascertain their potential for minimizing expression in nondopaminergic cells, and thereby reducing the likelihood of off-target effects. Achievement of this goal would be an important step forward in the development of Akt and hRheb for use in gene therapy applications in neurorestorative treatment of PD. Given the oncogenic potential of these molecules, we particularly sought to determine the use of these sequences to minimize expression in glial, as they are the predominant cellular source of primary human brain tumors. We have found that none of these sequences induced reporter expression in astrocytes in the SN. Admittedly some of the neuronal specificity achieved may have been due to the known neurotropic properties of AAV vectors. Nevertheless in our own experience with AAV 2/1 injections of the SN, we have occasionally observed glia to be transduced, particularly near the injection site. In addition, for reasons that are not clear, occasional expression is observed in glia for some particular transgenic proteins when the chicken beta actin promoter is used to mediate expression. Therefore, we believe that these hTHp sequences provide an additional margin of safety for avoiding transduction of glial cells. Furthermore, it may be possible in the future to achieve additional specificity of transduction by use of select or modified AAV serotypes.^11^

We also sought to determine if these sequences limited expression among neurons to the dopamine neurons of the SN. Both Akt and hRheb have biologic effects on nondopaminergic neurons,^12,13^ so expression of either molecule in adjacent nondopaminergic neurons could have off target effects such as disturbances in motor control due to altered function in these complex basal ganglia circuits. Injections of stratum and cortex with each of the hTHp sequences failed to induce any reporter expression, so in this assessment excellent specificity was achieved. However, surprisingly, within the SN itself, where patterns of expression were assessed by the identification of dopaminergic neurons by immunohistochemistry, specificity appeared to be much less, with 80% or more of the TagRFP-T-positive neurons not expressing TH. There are several possible reasons for this discrepancy. It is well known that immunohistochemistry does not detect all target antigens in tissue sections, due to limitations on the tissue penetration of protein reagents, and in particular to the entry of antibodies into the cell. Thus immunostaining will produce false negatives for the dopaminergic phenotype. In addition, we have observed that transduction of dopamine neurons with transgenes under either the chicken beta actin or the TH promoter can result in a suppression of expression of endogenous proteins, such as TH, used to identify the cell phenotype (personal observations). These considerations would not however explain the appearance of reporter expression in the SNpr where few dopamine neurons are located. These neurons are predominantly of the gamma-aminobutyric acid-ergic (GABAergic) phenotype. While TH is expressed in some GABAergic neurons of the brain, particularly those in the olfactory bulb,^14^ it is not known to be expressed in GABAergic neurons of the SNpr. Therefore, the reason why reporter is expressed in nondopaminergic neurons of the SNpr and yet not that of the striatum or cortex is unknown.

Among these hTHp sequences we did not identify an important difference in specificity. There was a trend for hTHp-522-TagRFP-T to be more specific but this difference was small. hTHp-522-TagRFP-T did however demonstrate a greater sensitivity for the detection of dopaminergic neurons; it successfully expressed reporter in 76% of the TH-positive neurons, whereas hTHp-5,3-TagRFP-T and hTHp-3.3-TagRFP-T achieved expression in only 33 and 40% of instances respectively.

We conclude that all of these hTHp sequences are capable of mediating expression in dopamine neurons *in vivo* with similar specificity in the SN. Because hTHp-522 as the smallest allows more space in the cassette for transgenes, and offers a higher level of sensitivity for achieving expression in SN dopamine neurons it may be the most useful in practical applications.

## Materials and Methods

### Production of AAV viral vectors

All vectors used for these studies were AAV1 serotype. A plasmid carrying a photostable fluorescent reporter TagRFP-T^15^ was obtained from Axxora (San Diego, CA). TagRFP-T DNA was amplified and cloned into an AAV backbone with the chicken β-actin promoter, and a woodchuck postregulatory element (WPRE) at the 3′ end (pBL). Three partial sequences of the hTHp were based on GenBank Acc# AY211521: 522bp (spanning from −491 to +31),^4^ a combination of 5′-flanking region (spanning from −509 to +1) and a 976 bp segment of 3′-flanking region DNA extending in the 3′ direction from the PolyA signal,^6^ and 3.3 kb (spanning from −3204 to +3)^7^ were produced by polymerase chain reaction amplification from human genomic DNA (Promega, Madison, WI) and subcloned in pGEM-T vector system (Promega).

The following primers pairs were used: for the 522bp sequence, 5′-AGACACACGGCCTGGAATCT-3′ (forward), 5′-CCTGTGGCGTGGTGGCGTCGGGGGTGGGCATGGCTCAGTGTGGA-3′ (reverse); and for the 3.3 kb sequence,5′-GACCAGTGTCTTGGGAGTTG-3′ (forward), 5′-CATGGCTCAGTGTGGAGGTCCGG-3′ (reverse). For the 5,3 combined promoter the 5′ flanking sequence was created from the 3.3 kb fragment by subcloning of its 3′terminus. Primers for the 3′ flanking region were based on GenBank Acc#NG_008128: 5′-TCACAATAAAAGAAACTGTGGTCT-3′ (forward), 5′-ACACCCCCAGCTGCAACCT-3′ (reverse). The chicken β-actin promoter in pBL-Tag-RFP-T was then replaced by each of these three partial sequences of hTHp to generate three different constructs: hTHp-522-TagRFP-T, hTHp-5,3-TagRFP-T, and hTHp-3.3-TagRFP-T. All nucleotide sequences in the AAV packaging constructs were confirmed before AAV production. AAVs were produced by the University of North Carolina Viral Vector Core (Chapel Hill, NC). The titer of AAV hTHp-522-TagRFP-T was 5 × 10^12^ viral genomes (vg)/ml; for AAV hTHp-5,3-TagRFP-T it was 1 × 10^12^ vg/ml, and for AAV hTHp-3.3-TagRFP-T it was 2 × 10^12^ vg/ml.

### Intracerebral AAV injections

Adult (8 weeks) males C57BL/6 were obtained from Charles River Laboratories (Wilmington, MA). Mice were anesthetized with ketamine/xylazine solution and placed in a stereotaxic frame (Kopf Instruments, Tujunga, CA) with a mouse adapter. For the injection of the SN the tip of a 5.0 µl syringe needle (26S) was inserted to stereotaxic coordinates AP: −0.35 cm, ML: +0.11 cm, DV: −0.37 cm (relative to bregma); for the striatum AP: +0.09 cm, ML: +0.22 cm, DV: −0.25 cm (relative to bregma); for the cortex AP: +0.09 cm; ML: +0.22 cm; DV: −0.125cm (relative to bregma). Viral vector suspension in a volume of 2.0 µl was injected at 0.1 µl/min over 20 minutes. Thus mice injected with hTHp-522-TagRFP-T, hTHp-5,3-TagRFP-T, and hTHp-3.3-TagRFP-T were injected with 1 × 10^10^, 2 × 10^9^, and 4 × 10^9^ vg. All injection procedures, as described above, were approved by the Columbia University Institutional Animal Care and Use Committee.

### Tissue processing

Post 6 weeks of AAV injections, mice were deeply anaesthetized and perfused intracardially with 0.9% NaCl followed by 4% paraformaldehyde in 0.1 mol/l phosphate buffer, pH 7.1. The brain was carefully removed and processed for sectioning on either a Vibratome (50 µm horizontal sections) or cryostat (30 µm coronal sections). For Vibratome sectioning the whole brain was postfixed 48 hours in 4% paraformaldehyde in 0.1 mol/l phosphate buffer, pH 7.1. For cryostat sectioning the brain was blocked into forebrain and midbrain regions. The forebrain was postfixed 48 hours; the midbrain was postfixed according to the specific antibody protocol (see below) and then cryoprotected overnight. Cryostat sections were cut after the brain was rapidly frozen by immersion in isopentane on dry ice. All sections were processed free-floating.

### Immuohistochemical staining procedures

Coronal cryostat sections containing SN or striatum (30 µm) and horizontal vibratome sections containing SN (50 µm) were immunostained either for TH (TH, 1:750, polyclonal, Calbiochem, Millipore, Billerica, MA) or calbindin (calbindin, 1:2,500, polyclonal, Swant, Marly, Swizerland) or AHD2 (AHD2, 1:300, polyclonal, Abcam, Cambridge, MA) or GFAP (GFAP, 1:800, monoclonal, Sigma, Ronkonkoma, NY). The midbrain was postfixed 48 hours for AHD2 and calbindin immunostaining, and 72 hours for GFAP staining. For staining with polyclonal antibodies sections were then treated with goat antirabbit AlexaFluor488. For GFAP staining with a monoclonal, sections were first treated with biotinylated antimouse IgG, followed by streptavidin AlexaFluor488. For some experiments, we performed a fluorescent Nissl counterstain (NeuroTrace 435/455 blue Fluorescent Nissl Stain, 1:150, Molecular Probes Thermo Fisher, Waltham, MA).

### Confocal imaging and quantification of TagRFP-T/Nissl-positive neurons, TH neurons, and TagRFP-T/Nissl/TH-positive neurons

Images of neurons expressing TagRFP-T and stained for Nissl and TH were acquired by confocal microscopy performed on 50 µm thick horizontal Vibratome sections through the SN. A Z-stack of images (20 optical planes of a 1 µm vertical distance) was obtained for SN ventral, SN dorsal, anterior, and posterior regions. The Argon-ion laser at 488 excitation wavelength was used to visualize the TH-positive neurons, the HeNe laser at 561 excitation wavelength to visualize TagRFP-T-positive neurons, and two-photon microscopy at 800 excitation wavelength for Nissl-positive neurons. For each hTHp construct, two mice were examined. From each mouse, two fields (515 × 515 µm) were obtained in the SNpc ventral, one field in the SNpr ventral, two fields in the SNpc dorsal anterior, two fields in the SNpc dorsal posterior, one field in the SNpr dorsal, for a total of eight fields. In each field, all TH-positive neurons, all TagRFP-T/Nissl-positive neurons and all TagRFP-T/Nissl/TH-positive neurons were counted. The percentage of TagRFP-T/Nissl/TH-positive neurons among the total number of TH-positive neurons (sensitivity) and the percentage of TagRFP-T/Nissl/TH-positive neurons among the total number of TagRFP-T/Nissl-positive neurons (specificity) were determined.

### Confocal imaging and quantification of TagRFP-T-positive neurons in the entire SN

Images of neurons expressing TagRFP-T and stained for TH were acquired by confocal microscopy performed on 30 µm thick coronal sections through the SN. Images were obtained from each of three rostro-caudal planes of the SN: anterior to the medial terminal (MT) nucleus of the accessory optic tract, containing the MT and posterior to the MT. The Argon-ion laser at 488 excitation wavelength was used to visualize the TH-positive neurons and the HeNe laser at 561 excitation wavelength was used to visualize TagRFP-T-positive neurons. Four mice for each hTHp-5,3-TagRFP-T and hTHp-3.3-TagRFP-T, 12 mice for hTHp-522-TagRFP-T were examined. Seven fields (775 × 775 µm) spanning the entire SN was acquired from each mouse, with each field slightly overlapping adjacent fields. Based on these images the SN and its borders, based on the TH staining were reconstructed by use of Adobe Photoshop. All TagRFP-T-positive neurons were counted in the delineated area. The total number of TagRFP-T-positive neurons within the three SN planes was determined and compared among the three hTHp constructs.

### Statistical analysis

Differences between the three constructs were analyzed by one-way analysis of variance and Tukey’s *post hoc* analysis. All statistical analyses were performed using SigmaStat software (version 12) (Systat Software, San Leandro, CA).

## Author Contributions:

Experiments were designed by A.S.R., N.K., and R.E.B. Experiments were performed by A.S.R., T.K., O.Y., and N.K., A.S.R., N.K., and R.E.B. wrote the manuscript.

## Figures and Tables

**Table 1:** Sensitivity and specificity of the three partial sequences of human TH promoter (hTHp) for TH-positive neurons in the SN

*AAV construct (6 weeks postinjection)*		*hTHp-522-Tag-RFP-T*	*hTHp-5,3-Tag-RFP-T*	*hTHp-3.3-TagRFP-T*
Sensitivity				
	SN total	76%	33%	40%
	SNc ventral	79%	21%	37%
	SNc dorsal	74%	38%	40%
	SNr	93%	43%	55%
Specificity				
	SN total	20%	17%	18%
	SNc ventral	19%	15%	17%
	SNc dorsal	25%	24%	27%
	SNr	2%	3%	1%

AAV, adeno-associated viral; SN, substantia nigra; TH, tyrosine hydroxylase.

To determine the sensitivity and specificity of each promoter sequence for expression in TH-positive dopamine neurons, the relationship between TagRFP-T expression and TH immunostaining was determined for each. In order to facilitate the detection of TagRFP-T expression in neurons, given its punctate nature, the sections were counterstained with a fluorescent Nissl stain, and TagRFP-T expression was counted only if detected in a Nissl-positive neuronal profile. Immunostaining for TH was then visualized and the number of TH-positive neurons positive or negative for TagRFP-T was determined for each of the regions specified. Among the three constructs, the 522 bp sequence of the human TH promoter provides the highest sensitivity and specificity for all considered regions. Two mice were studied for each sequence, and among the six mice 11,107 neurons were assessed.
